# Supplementary material for: Insights into the State of the Art of Urogenital Schistosomiasis with a Focus on Infertility
Source: Trop Med Infect Dis. 2024 Aug 10;9(8):177. doi: 10.3390/tropicalmed9080177 (PMC11360082; doi:10.3390/tropicalmed9080177)
Supplement: Supplementary file 1 [file tropicalmed-09-00177-s001.zip › tropicalmed-3012065-supplementary.pdf]

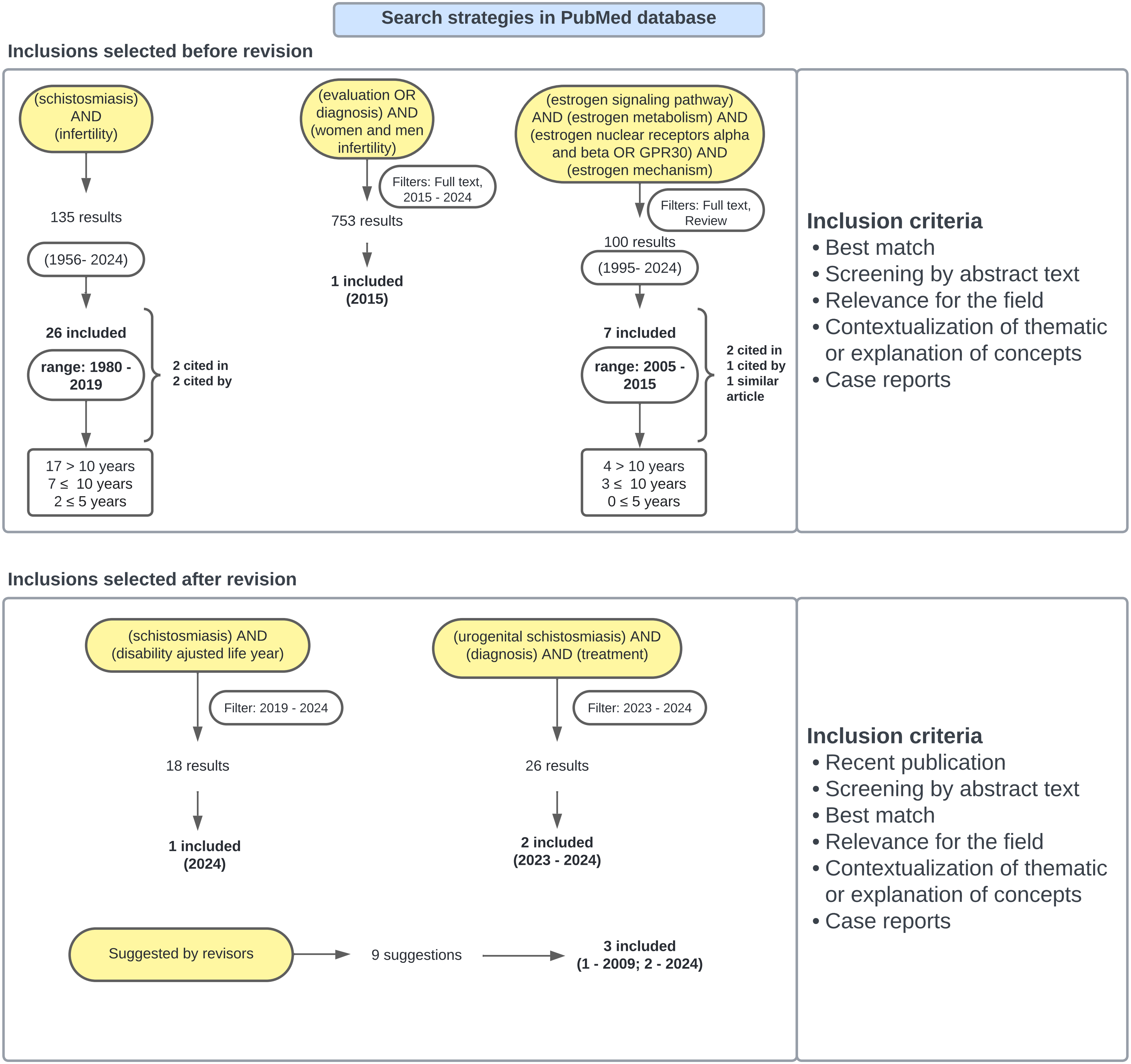
Search strategies in PubMed database
Inclusions selected before revision
(schistosmiasis) AND (infertility)
135 results
(1956- 2024)
26 included
range: 1980 - 2019
2 cited in
2 cited by
17 > 10 years
7 ≤ 10 years
2 ≤ 5 years
(evaluation OR diagnosis) AND (women and men infertility)
Filters: Full text, 2015 - 2024
753 results
1 included (2015)
(estrogen signaling pathway) AND (estrogen metabolism) AND (estrogen nuclear receptors alpha and beta OR GPR30) AND (estrogen mechanism)
Filters: Full text, Review
100 results
(1995- 2024)
7 included
range: 2005 - 2015
2 cited in
1 cited by
1 similar article
4 > 10 years
3 ≤ 10 years
0 ≤ 5 years
Inclusion criteria
• Best match
• Screening by abstract text
• Relevance for the field
• Contextualization of thematic or explanation of concepts
• Case reports
Inclusions selected after revision
(schistosmiasis) AND (disability ajusted life year)
Filter: 2019 - 2024
18 results
1 included (2024)
(urogenital schistosmiasis) AND (diagnosis) AND (treatment)
Filter: 2023 - 2024
26 results
2 included (2023 - 2024)
Suggested by revisors
9 suggestions
3 included (1 - 2009; 2 - 2024)
Inclusion criteria
• Recent publication
• Screening by abstract text
• Best match
• Relevance for the field
• Contextualization of thematic or explanation of concepts
• Case reports
